# Correction of Body-Mass Index Using Body-Shape Perception and Socioeconomic Status in Adolescent Self-Report Surveys

**DOI:** 10.1371/journal.pone.0096768

**Published:** 2014-05-20

**Authors:** Stéphane Legleye, François Beck, Stanislas Spilka, Nearkasen Chau

**Affiliations:** 1 Institut national d’études démographiques (INED), Paris, France; 2 Inserm, U669, Univ Paris-Sud and Univ Paris Descartes, UMR-S0669, Paris, France; 3 Institut national de prévention et d’éducation à la santé (INPES), Saint-Denis, France; 4 Cermes3 - Equipe Cesames (Centre de recherche Médecine, Sciences, Santé, Santé mentale, Société, Université Paris Descartes/CNRS UMR 8211/Inserm U988/EHESS), Paris, France; 5 French Monitoring Centre for Drugs and Drug Addiction - Observatoire Français des Drogues et des Toxicomanies (OFDT), Saint-Denis, France; University of Missouri-Kansas City, United States of America

## Abstract

**Objectives:**

To propose a simple correction of body-mass index (BMI) based on self-reported weight and height (reported BMI) using gender, body shape perception and socioeconomic status in an adolescent population.

**Methods:**

341 boys and girls aged 17–18 years were randomly selected from a representative sample of 2165 French adolescents living in Paris surveyed in 2010. After an anonymous self-administered pen-and-paper questionnaire asking for height, weight, body shape perception (feeling too thin, about the right weight or too fat) and socioeconomic status, subjects were measured and weighed. BMI categories were computed according to Cole’s cut-offs. Reported BMIs were corrected using linear regressions and ROC analyses and checked with cross-validation and multiple imputations to handle missing values. Agreement between actual and corrected BMI values was estimated with Kappa indexes and Intraclass correlation coefficients (ICC).

**Results:**

On average, BMIs were underreported, especially among girls. Kappa indexes between actual and reported BMI were low, especially for girls: 0.56 95%CI = [0.42–0.70] for boys and 0.45 95%CI = [0.30–0.60] for girls. The regression of reported BMI by gender and body shape perception gave the most balanced results for both genders: the Kappa and ICC obtained were 0.63 95%CI = [0.50–0.76] and 0.67, 95%CI = [0.58–0.74] for boys; 0.65 95%CI = [0.52–0.78] and 0.74, 95%CI = [0.66–0.81] for girls. The regression of reported BMI by gender and socioeconomic status led to similar corrections while the ROC analyses were inaccurate.

**Conclusions:**

Using body shape perception, or socioeconomic status and gender is a promising way of correcting BMI in self-administered questionnaires, especially for girls.

## Introduction

Obesity is responsible for numerous health complications, chronic diseases and increased risk of mortality [1], and it contributes to health inequalities because it mainly concerns poor families [2]. Its prevention and treatment account for a large share of health budgets in Western countries (1.5%–4.6% of the annual expenditures in France [3]). Obesity occurs very early in life and has psychological and social consequences among young people, such as discrimination [4] and bullying [5]. Monitoring the adolescent population is thus an important public health objective, especially because there are indications in some countries that the social gradient in childhood obesity may be increasing over time [6].

The most widely-used indicator of obesity is the Body Mass Index (BMI) based on height and weight [7], although other indicators may be more reliable [8]. Unfortunately, employing technicians to measure these data is often impossible in large-scale surveys, and alternative indicators such as waist circumference and waist-to-hip ratio require a certain amount of training to be reliable.

Further to this, “reported” BMI (based on self-reported height and weight) is not reliable for estimating the true prevalence of obesity in a population. In adult populations, under-reporting for weight and BMI and over-reporting for height is common, although the extent of under-reporting varies between men and women [9]. For example, in a representative sample collected in the general population of France in 2002–2003, on the basis of self-report, 32.2% of subjects who were actually obese were misclassified as non-obese (BMI<30) whereas only 0.9% of actually non-obese individuals were misclassified as obese [10], showing that there was a strong BMI-related bias in these reports; a more recent analysis conducted in 2006–2007 reported a similar bias [11]. Other biases also exist, such as the occupational category [12], socioeconomic status [13], age and ethnic origin [14]. In an adolescent population, a recent literature review indicated that the sensitivity of reported BMI for screening for actual overweight ranged from 55% to 76%, and that reported overweight prevalence was 0.4% to 17.7% lower than actual prevalence [15]. As among adults, there was a strong weight-related bias in the underreporting of weight.

Efforts have therefore been made to remedy this situation and correct reported height and weight by means of additional information such as age, gender, reported diabetes mellitus or smoking habits [16], waist-to-hip ratio, health status, or health-care variables [17]. This is difficult to generalise and requires a battery of measures or variables. Following a different approach, a recent publication [18] provided new thresholds for screening for obesity with self-reported measures, but only in the adult population, in Switzerland. The threshold found cannot therefore be used in adolescents or young adults.

Besides gender, two major characteristics are linked to the bias in reported BMI: social status [19], [20] and actual weight. The assessment of the social status of their parents by adolescents is often difficult: it requires numerous questions, and responses are frequently missing or inaccurate because of ignorance, misunderstanding or a desirability bias, but it is nevertheless generally judged sufficiently reliable [21]. Its association with the bias in reported BMI seems not to be systematic in adolescence [11], [22]. Regarding weight, it is by definition unknown, but, as shown in [23], one way of bypassing this difficulty may be to use body shape perception because it helps to predict the bias in self-reported weight: adolescents who regard themselves as too fat may more readily underestimate their BMI [22], whereas people satisfied with their body image are less prone to under-reporting their weight [17]. This approach is all the more promising because the social norms for thinness differ across social classes, gender and age groups [17], [24]. As noted by de Saint-Pol [25], the “ideal” BMI, defined as the BMI that represents a balance of judgments on one’s body shape, is lower among women than men and is lower among higher social categories: body dissatisfaction and desire for slimness are common in high –socio-economic environments across the world [26]. Using body perception could thus be efficient for correcting BMI. This approach has been successfully used in a representative German adolescent population [27]. It is also supported by a recent French study on adolescents exploring a wide range of socioeconomic and health-related factors, which showed that BMI under- or over-reporting (compared with measured data) were mainly influenced by age, gender, the father’s occupation, actual BMI, and body image perception [28].

More generally, if BMIs were adequately corrected, epidemiologists would be able to estimate the prevalence of underweight, over-weight, obesity, and normal weight. This would be useful to obtain a more accurate picture of the distribution of body shapes in the population as well as to provide early warning to screen for anorexia nervosa (which concerns around 0.3% and 0.9% of adolescents [29], [30]) in large-scale surveys.

The aim of this study is to propose strategies that could be easily tested in different populations in order to compute corrected BMI from self-administered adolescent surveys. They are based on the use of gender and a variable related to body shape perception (BSP) or socioeconomic status (SES).

## Methods

### Sample and Protocol

The ESCAPAD survey (Survey on health and behaviour) is regularly carried out by the French Monitoring Centre for Drugs and Drug Addiction with the National Service department during the national defence preparation day (JAPD). Attendance at this one-day session of civic and military information is compulsory for all French adolescents when they reach their 17^th^ birthday. The ESCAPAD survey takes place in March in all the 300 civilian or military centres across the country. Participants are guaranteed complete confidentiality and anonymity and the completion of the pen-and-paper self-administered form is entirely voluntary: this is explicitly stated by the staff before the distribution of the questionnaire. The survey has gained the Public Statistics general interest seal of approval from the National Council for Statistical Information (2008X713AU) as well as the approval of the ethics commission of the National Data Protection Authority (CNIL). A complete description has been published elsewhere [31], [32].

In 2010, a specific ESCAPAD survey was conducted in the city of Paris (n = 2,165) from 6^th^ October to 6^th^ December. The questionnaire was completed in the morning. Adolescents attending the day (whether or not they completed the questionnaire) were informed that a random sample would attend an additional face-to-face interview in the afternoon, but this announcement did not mention that they would be measured and weighed during the session. The four interviewers in the afternoon were members of the Survey and Sampling Department of the National Institute for Demographic Studies (INED: www.ined.fr), and all are specialists in qualitative research and interviewing on sensitive topics. Training was organised at INED and work meetings were conducted each week.

### Ethics

Based on an examination of the protocol and questionnaire of the Paris survey, the approval of the CNIL did not require written consent of the participants nor that of the parents of minors over 16 years old.

### Questionnaire

Reported height and weight (in centimetres and kilograms) were used to compute reported BMI. Body shape perception –BSP- (“How do you feel about your body? “much too thin”, “a bit too thin”, “about the right weight”, “a bit too fat”, “much too fat”) was recoded in three categories by combining the upper and lower response categories, giving too thin, about the right weight, too fat. This question is commonly used this way in studies on obesity and mental health to identify adolescents who “feel fat” [33] or (using a simple dichotomisation) to identify adolescents who perceive themselves as overweight [34].

Socioeconomic status – SES - was based on the report of the exact occupation of each parent coded according to the national typology [35]. We used the highest category (in this order: 1. managers/professionals; 2. intermediate professions; 3. self-employed; 4. white collars workers; 5. manual workers; 6. farmers; 7. inactive/unemployed) to compute a synthetic 3-level SES variable (high SES = 1; middle SES = 2,3; low SES = 4,5,6,7). Missing values (10%) were all handled by professional coders before the analysis. This coding has been used in other studies based on this survey [36]–[38].

### Material and Setting

The interviewers were trained in the protocol and use of equipment [39]. The scales were electronic s with automatic calibration and a precision of 0.1 kg which was checked regularly. The height gauges (precision 0.5 cm) were fixed to the wall: a small chair was used to read the height of the tallest individuals. Before the measurements, the adolescents were asked to remove their jackets, pullovers, watches, jewelry and shoes and to empty their pockets. A correction of 0.6 kg for the weight of the remaining clothes during weighing was applied. The references for BMI categories were taken from the study by Cole et al. [40], [41].

### Statistical Analysis

Differences in categorical (resp. continuous) variables were tested with Pearson Chi^2^ tests (resp. by t-tests). Two models based on linear regressions were used to correct reported BMI, where names in italic are dummy binary variables coding for BSP (resp. SES) categories and ε is a random term.

Model 1:
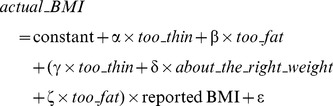



Model 2:
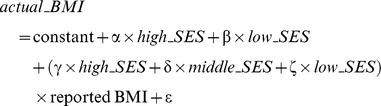



All regressions were computed separately for boys and girls. The quality and predictive power of each model was assessed using R^2^ and the Root mean square of errors (RMSE). These analyses were first conducted on the full sample with no missing values: a cross validation was then conducted using the leave-one-*out* method and the resulting RMSE values were computed (the lower, the better). Then, missing values for actual and reported BMI (7.6%) and BSP (1.5%) were handled by multiple imputation regressions using the Monte Carlo Markov chain for BMI and logistic regression for BSP: 5 imputations were produced this way [42] to compute regression coefficients. The impact of non-response was assessed by comparing the sets of coefficients obtained in the respondent and imputed datasets (the closer, the better). SES had no missing values.

The final indicator of the accuracy of the models was the agreement between the categories of corrected BMI and actual BMI. For this purpose, we computed weighted Kappa indexes [43] and Intraclass correlation coefficients (ICC) with 95% confidence intervals.

Finally, as in the work by Dauphinot et al. [18], ROC analyses (receiver operating characteristics) [44] were used, to provide a corrected threshold for obesity, overweight and underweight for the two genders. This analysis was computed only on the initial dataset without imputing the missing values. We then applied the corrections to the whole Paris sample in the ESCAPAD survey (n = 2,165, age = 17, 18) from which the present subsample of individuals was taken. The significance level was set at 0.05. All analyses were conducted using SAS V9.3.2.

## Results

The initial random sample comprised 176 boys and 165 girls aged 17–18, of whom 85.4% were still at school, 6.6% were in a vocational training, and 8.0% were unemployed or working. This is consistent with the results found in the whole Paris sample.

### Non-response

In all, 7.6% of the individuals refused to report their height or weight (n = 26), with a non-significant difference between boys and girls (6.3% vs 9.1%, p = 0.323). Among them, 5.0% refused to measure their height, while 6.5% refused to report their weight (without significant difference between genders), and 5.3% refused to measure both their height and weight (n = 18), girls more often than boys (8.5% vs 2.3%, p = 0.010). In the whole sample, both reported and measured BMI was obtained for 88.9%. Only 1.5% (5 subjects) refused to answer the question concerning their BSP and their reported BMI was also missing. There were no missing values for SES. The final sample represented 88.5% of the initial sample: it comprised 163 boys and 140 girls with no missing values for BSP, SES, actual and reported height and weight. The imputed dataset comprised 174 boys and 161 girls (6 individuals with missing actual and reported BMIs were removed).

### Validity of Reported BMI

Table 1 shows that 16.3% of the subjects found themselves (much) too thin (23.3% of the boys, 7.9% of the girls) while 24.1% found themselves (much) too fat (14.7% of the boys, 35.0% of the girls). Using Cole’s criteria, the proportions of overweight/obese individuals using reported BMI were much lower than those obtained using actual BMI, especially among girls (6.4% and 1.4% vs 12.9% and 3.6%). In addition, girls tended to present themselves as thin (16.4% instead of 5.7% for the actual BMI).

**Table 1:** Actual and reported BMI by gender (mean, standard deviation –sd, and Cole’s classification).

	Boys N = 163	Girls N = 140	Difference between genders: P-value
Mean reported height (sd)	179.52 (6.38)	166.12 (6.38)	0.001
Mean actual height (sd)	178.16 (6.62)	164.86 (6.30)	0.001
Mean difference (sd)	−1.36 (3.04)	−1.26 (2.24)	0.755
Mean reported weight (sd)	69.66 (10.56)	57.39 (8.95)	0.001
Mean actual weight (sd)	70.63 (11.75)	59.75 (9.72)	0.001
Mean difference (sd)	1.02 (5.25)	2.36 (3.44)	0.010
Mean reported BMI (sd)	21.56 (2.65)	20.82 (3.29)	0.033
Mean actual BMI (sd)	22.21 (3.10)	22.02 (3.74)	0.636
Mean difference (sd)	0.65 (1.79)	1.21 (1.50)	0.003
Reported BMI classification (%)			
Underweight	8.0	16.4	0.030
Normal weight	81.0	75.7	
Overweight	11.0	6.4	
Obese	0.0	1.4	
Actual BMI classification (%)			
Underweight	6.1	5.7	0.378
Normal weight	74.9	77.9	
Overweight	17.8	12.9	
Obese	1.2	3.6	
Agreement: reported vs actual BMI			
Weighted Kappa	0.56	0.45	–
(95%CI)	(0.42–0.70)	(0.30–0.60)	
ICC	0.58	0.56	–
(95%CI)	(0.47–0.67)	(0.44–0.66)	
% of correctly classified (%)	83.4	75.0	
Body shape perception (%)			
(Much) too thin	23.3	7.9	0.001
About the right weight	62.0	57.1	
(Much) too fat	14.7	35.0	
Objective socioeconomic status (%)			
Low	39.3	32.1	0.092
middle	31.3	26.5	
high	29.4	41.4	
Subjective socioeconomic status (%)			
Over average	33.1	35.0	0.998
Around average	60.7	59.3	
Below average	6.1	5.7	

Reported BMI is based on self-reported weight and height.

Source: Escapad Paris 2010, analytic subsample without non-respondents (n = 303).

The agreement between reported and actual BMI categories was moderate for boys (kappa = 0.56, 95%CI = [0.42–0.70]; ICC = 0.58, 95%CI = [0.47–0.67]; percentage of correctly classified individuals = 78.9%), and low among girls (kappa = 0.45, 95%CI = [0.30–0.60]; ICC = 0.56, 95%CI = [0.44–0.66]; 75.0%). However, the Pearson’s correlation coefficients between reported and actual BMIs as continuous variables were high: 0.82 among boys, 0.92 among girls.

### Regression Analyses

In the raw dataset (Table 2), the comparison of the slopes γ, δ and ζ showed they were generally different, and differed between boys and girls. These results suggest the need for separate analyses by gender. The R^2^ values were lower for boys than girls, whereas the RMSE values were higher for boys, showing that the models are more efficient among girls. The lowest RMSE was obtained for model 1 (BSP) for boys and girls. The cross-validation RMSE values were only slightly above the initial values and the comparison of the coefficients obtained in the raw and imputed datasets shows that the results are similar, especially for the slopes γ, δ, ζ. These last two findings suggest that the results are robust.

**Table 2:** Regression coefficients of reported BMI in three models.

	Raw dataset		Imputed dataset	
	Boys N = 163	Girls N = 140	Boys N = 174	Girls N = 161
***Correction by body-shape perception: model 1***				
Constant	2.17	1.21	1.63	1.46
α for(much) too thin	6.68	19.33	6.72	19.01
β for (much) too fat	−5.92	0.78	−3.13	0.31
γ for (much) too thin × reported BMI	0.57	−0.12	0.59	−0.12
δ for about the right weight × reported BMI	0.92	0.99	0.95	0.98
ζ for (much) too fat × reported BMI	1.21	0.99	1.11	1.00
R^2^	0.70	0.87		
RMSE	1.70	1.35		
Cross-validation RMSE	1.83	1.40		
***Correction by objective socioeconomic status: model 2***				
Constant	2.47	−2.66	2.54	−1.73
α for Low	−2.60	2.18	−2.78	0.89
β for High	−0.06	4.58	−1.17	3.09
γ for low × reported BMI	1.03	1.06	1.04	1.08
δ for middle × reported BMI	0.92	1.21	0.92	1.16
ζ for high × reported BMI	0.91	0.97	0.96	1.00
R^2^	0.67	0.85		
RMSE	1.80	1.47		
Cross-validation RMSE	1.88	1.51		

Reported BMI is based on self-reported weight and height;

Source: Escapad Paris 2010, analytic subsample with non-respondents (n = 341).

Table 3 shows mean corrected BMI and categories in the non-imputed dataset. Compared to the situation with uncorrected data, the agreement of the actual and the corrected BMI categories using model 1 (BSP) was improved among boys (Kappa = 0.63 95%CI = [0.50–0.76]; ICC = 0.67, 95%CI = [0.58–0.74]) and especially among girls (Kappa = 0.65, 95%CI = [0.52–0.78]; ICC = 0.74, 95%CI = [0.66–0.81]). Agreement tended to be better for girls than boys and better in model 1 (BSP) among boys but better in model 2 (SES) among girls. In particular, no obese boy was successfully classified in model 2 (SES).

**Table 3:** Corrected BMI (mean, standard deviation –sd, Cole’s classification, Kappa and Intraclass correlation coefficients indexes with 95% confidence intervals −95%CI).

	Boys	Girls
***Correction by body shape perception: model 1***		
Mean corrected BMI (sd)	22.21 (2.62)	22.02 (3.49)
Mean corrected BMI-actual BMI (sd)	−0.00 (1.68)	−0.00 (1.33)
Corrected BMI classification (%)		
Underweight	3.7	7.1
Normal weight	79.1	80.0
Overweight	16.0	10.7
Obese	1.2	2.1
Agreement with actual BMI		
Weighted Kappa	0.63	0.65
(95%CI)	(0.50–0.76)	(0.52–0.78)
ICC	0.67	0.74
(95%CI)	(0.58–0.74)	(0.66–0.81)
% of correctly classified (%)	85.3	85.0
***Correction by objective socioeconomic status: model 2***		
Mean corrected BMI (sd)	22.21 (2.55)	22.02 (3.45)
Mean corrected BMI-actual BMI (sd)	−0.00 (1.78)	−0.00 (1.78)
Corrected BMI classification (%)		
Underweight	4.3	5.0
Normal weight	81.0	82.1
Overweight	14.7	10.7
Obese	0.0	2.1
Agreement with actual BMI^a^		
Weighted Kappa	0.58	0.68
(95%CI)	(0.44–0.72)	(0.55–0.82)
ICC	0.59	0.76
(95%CI)	(0.48–0.68)	(0.68–0.82)
% of correctly classified (%)	83.4	87.1

a overweight and obese are aggregated for boys.

Source: Escapad Paris 2010, analytic subsample without non-respondents (n = 303).

Considering results of model 1 as the most balanced for boys and girls, we found that the proportion of underweight girls based on reported BMI was reduced by 57%, while the proportions of overweight and obese girls increased by 67% and 50% and were much closer to the actual values (but still underestimated). The corrected proportion of underweight boys was still below actual values, but the proportion of overweight or obese boys was close to the actual values.

We also conducted a ROC analysis (in the dataset without imputation) to compute the optimal thresholds for reported BMI to screen for obesity, underweight, and overweight. For obesity screening among boys, the best threshold was 27.7 for reported BMI, the corresponding sensitivity (Se) and specificity (Sp) were 100.0% and 98.8%, and the AUC (area under ROC curve) was 0.99 95%CI = [0.97–1.00]. For girls, the results were 24.1, Se = 83.3%, Sp = 91.8% and AUC = 0.97 95%CI = [0.93–1.00]. Using the same procedure, the optimal threshold for overweight was 23.4 among boys (Se = 81.8, Sp = 91.6), and 22.5 among girls (Se = 84.0, Sp = 89.1); the optimal threshold for underweight was 19.8 among boys (Se = 83.3, Sp = 75.0) and 19.4 among girls (Se = 75.0, Sp = 63.3). However, when the optimal thresholds for all BMI categories were combined, the Kappa indexes and percentages of correctly classified individuals were not as good as with models 1 and, 2, especially for girls: Kappa = 0.52 95%CI = [0.41–0.63], percentage of correctly classified = 69.3% for boys; Kappa = 0.41 95%CI = [0.31–0.51], percentage of correctly classified = 51.5% for girls. One reason was the poor correction for obesity, especially among girls: the procedure led to corrected proportions of “obese” individuals that were 2.5% instead of 1.2% among boys and 11.4% instead of 3.6% among girls.

Table 4 shows the estimated prevalences of corrected BMI categories when applying models 1 and 2 to the whole Paris sample from the ESCAPAD survey (n = 2,165). The effect on BMI average, underweight and overweight/obese BMI categories was considerable, especially among girls. For example, using model 1 (with BSP), the mean corrected BMI was 22.04 instead of 21.34 among boys and 21.57 instead of 20.29 among girls. The corrected proportions of overweight and obese were 10.5% and 3.2% among boys (compared to 7.8% and 1.9% before correction) and the corresponding values were 9.8% and 1.9% among girls (compared to 4.3% and 1.2% before correction).

**Table 4:** Estimated BMI and BMI categories in the Paris sample (mean, standard deviation –sd, and Cole’s classification).

	Boys	Girls
*Body shape perception (%)*		
(Much) too thin	20.6	7.7
About the right weight	63.8	57.0
(Much) too fat”	15.7	35.3
*Objective socioeconomic status (%)*		
Low	26.8	28.8
Middle	24.2	21.8
High	49.0	49.3
*Reported BMI*		
Mean (sd)	21.34 (2.76)	20.29 (2.85)
BMI classification (%)		
Underweight	8.3	22.7
Normal weight	82.0	71.8
Overweight	7.8	4.3
Obese	1.9	1.2
***Correction by body-shape perception: model 1***		
Mean corrected BMI (sd)	22.04 (2.90)	21.57 (3.02)
Mean difference (sd)	0.69 (0.57)	1.28 (0.64)
Corrected BMI classification (%)		
Underweight	1.9	7.1
Normal weight	84.4	81.2
Overweight	10.5	9.8
Obese	3.2	1.9
***Correction by objective socioeconomic status: model 2***		
Mean height and weight corrected BMI (sd)	21.97 (2.64)	21.47 (3.04)
Mean difference (sd)	0.63 (0.22)	1.18 (0.41)
BMI classification (%)		
Underweight	3.1	8.1
Normal weight	83.5	81.9
Overweight	11.3	7.9
Obese	2.1	2.0

Source: Escapad Paris 2010, whole sample: n = 2165, age = 17,18.

## Discussion

This study aimed to propose a simple correction of BMI obtained in a self-administered adolescent survey, using only self-reported BSP or SES as external information. Two linear models were used to correct reported BMI: 1/based on body-shape perception (BSP); 2/based on socioeconomic status (SES). The robustness of the corrections was evaluated through a cross-validation and multiple imputations. Model 1 gave the best and most balanced Kappas and ICCs for both genders (Kappa = 0.63, ICC = 0.67 for boys, Kappa = 0.65 and ICC = 0.74 for girls). Both strategies improved the estimation of BMI for both genders, and especially for girls. Using model 2 (SES) instead of model 1 (BSP) led to an overestimation of numbers of underweight boys and an underestimation of numbers of obese boys. For girls, the main difference between model 1 and 2 was that model 1 rated more girls as underweight, but the Kappas and ICCs were very close. By comparison, a ROC analysis used to determine the optimal thresholds of reported BMI for screening actual underweight, overweight and obesity yielded less accurate results.

### Comparison with other Studies

Most studies focus on comparing reported and measured BMI in order to determine which characteristics most influence the reporting bias, some introducing numerous variables into the analysis [16], [17]. Despite a greater potential accuracy, this strategy produces less reproducible studies, as the number of additional variables would need to be large in order to correct the values reported in self-administered questionnaires. The method used here is comparatively more parsimonious and easier to implement [45]. ROC analyses based on the study by Dauphinot et al. [18] have also been applied to determine the optimal cut-offs for reported BMI that predicts real obesity. The fact that our ROC analyses led to inaccurate results may be due to the restricted numbers of subjects, especially obese subjects, but also to the fact that it did not consider auxiliary variables.

Our actual and corrected BMI categories can be compared to other French studies. In a regional sample of schoolchildren aged 6–11 (n = 1000) surveyed in 2004 in the south of France, the prevalence of overweight and obesity also based on Cole’s criteria were found to be 17.3% and 3.3%, respectively, with similar prevalences in boys (15.8%, 2.9%) and girls (18.8%, 3.7%) [46]. Using a national sample of adolescents aged 14–17, Lobstein and Frelut found that measured overweight/obesity was around 16% in France in 2003, according to Cole’s BMI categories [47]. For 11–14 year-olds, the prevalences of reported for overweight and obesity were found to be 13.1% and 2.1% in 2006–2007, with no significant differences between genders [48]. In a national representative study of pupils aged 11–15 years conducted in 2006, prevalence of reported overweight/obesity was similar [49], while a representative school survey in one French eastern region found higher actual obesity prevalence, and under-reporting was twice as common as over-reporting [28].

These differences in overweight/obesity prevalence can be explained by differences in age, regional dietary and lifestyle habits and socioeconomic status [28], [49]. But overall, no substantial change in the prevalence of overweight and obese children and adolescents was noted in France between 2000 and 2007, this stability being partly due to large-scale health and obesity prevention campaigns in the context of the National Nutritional Health Programme (*Programme National Nutrition-Santé*), according to certain authors [50]. Results concerning underweight are comparatively scarce: in a sub-sample of 83 18–29 year-olds taken from a national survey among 18–74 years old conducted in 2006–2007, Julia et al. found that 8.4% were underweight (actual BMI<18.5) [11], that is close to results in table 4.

### Limitations

First, our sample size is small, so its statistical power is limited. This is particularly true for the extreme categories of BSP. In these categories, the regression statistics suggest that the correction strategies are rather ineffective. A larger sample would be required to confirm whether this result is due to our small number of subjects or rather reflects particular individual variability. Second, the procedure was limited to subjects aged 17–18 years old who were interviewed using a pen-and-paper self-administered questionnaire. Different results might also be obtained if a different data collection mode were used or if an interviewer was to be included in the process [51].

More importantly, our method is based on the assumption that social background (or parental social status) (SES) and body-shape perception (BSP) are confounders of the bias in self-reported BMI for the two genders because of related social body norms. Self-reported social background is subject to bias, such as social desirability or ignorance regarding the parents’ occupation, but it can be considered independent from BMI. This may not be the case for BSP which could vary with SES even when actual BMI is controlled for. We checked that no interaction of this kind was significant, as found by [52].

Nevertheless, the use of our item regarding BSP raises some questions. It is exactly the same as the one used in [33], [34]; it is related to “feeling fat” rather than to “being fat” and we used the answer “feeling (much) too fat” as a proxy for the perception of overweight, as in the study by Perrin et al. [34]. As underlined by Allen et al. [53], there are differences between over-concern with weight and shape and body dissatisfaction, which our measure of “feeling fat” tends to mix together. But unlike most of the studies that either aimed to disentangle the components of body image or tried to quantify the effects of each of these components on the mental health, we were only interested in the corrective potential of this subjective measure.

Perrin et al. [34] found that the perception of true overweight varies with actual BMI: the proportion of adolescents who perceived themselves as overweight was positively linked with the BMI percentile category and was highest among the actually overweight individuals, especially among girls. For boys, this proportion ranged from 2.7% among those in the 0<60% BMI category, to 23.9% among those in the 75%–85% BMI category and finally to 60.9% among those in the ≥85% BMI category (i.e. overweight). For girls, the corresponding values were: 3.0% among those in the 0–20% BMI category, to 46.5% among those in the 60%–85% BMI category and finally 82.4% among those in the ≥85% BMI category. This clearly supports the fact that a correct perception of overweight is much more likely among those who are actually overweight. Our correction strategy is based on this result. Nevertheless, the study by Perrin et al. shows that a large proportion of the girls misperceived their shape, as they (wrongly) thought they were overweight, especially among those in the 60–85% BMI category (46.5%). Our correction strategy may therefore lead to inaccurate corrections among normal-weighted girls, in particular among those whose BMI is close to the overweight category.

This finding should be related to the fact that, as found in [54], [55], girls are more influenced by the media, parents and peers than boys to engage in strategies to lose weight. For them, body dissatisfaction, body importance and the feeling of being fat are more markedly the result of social pressures. It is because of these worries about weight and shape that the correction of BMI is better for girls than boys, despite some inaccurate corrections for normal-weighted girls. The fact that the correction is less efficient among boys could also be related to the fact that for them, muscles may to some extent have greater importance than fat [56], [57]. Incorporating a measure of body dissatisfaction linked to the perceptions of muscle would be interesting.

## Conclusions

Using body shape perception and objective socioeconomic status is a promising way of correcting BMI based on reported height and weight. Replications of this study are needed.
